# Association of serum follistatin levels with histological types and progression of tumor in human lung cancer

**DOI:** 10.1186/s12935-018-0664-2

**Published:** 2018-10-20

**Authors:** Pengyu Zhang, Yingxin Ruan, Jun Xiao, Fangfang Chen, Xuejun Zhang

**Affiliations:** 1 Department of Clinical Laboratory, Tianjin Medical University Cancer Institute and Hospital, National Clinical Research Center of Cancer, Key Laboratory of Cancer Prevention and Therapy, Tianjin, 300060 People’s Republic of China; 2 Department of Nephrology, General Hospital of Tianjin Medical University, Tianjin, 300052 People’s Republic of China; 3 Department of Gastrointestinal Colorectal and Anal Surgery, China-Japan Union Hospital of Jilin University, Changchun, 130031 People’s Republic of China; 4 Department of Immunology, Key Laboratory of Educational Ministry of China, Tianjin Key Laboratory of Cellular and Molecular Immunology, School of Basic Medical Sciences, Tianjin Medical University, No. 22 Qixiangtai Road, Heping District, Tianjin, 300070 People’s Republic of China

**Keywords:** Follistatin, Small cell lung cancer, Non-small cell lung cancer, Histological types

## Abstract

**Background:**

Follistatin (FST), an activin-binding protein, inhibits activin action by interfering with activin binding to its receptor. The prognostic value of FST has been studied in various cancers. However, these studies rarely focus on lung cancer. In our study, we investigated the relationship between serum FST levels and lung cancer with histologic types, TNM staging, and recurrence.

**Methods:**

A total of 150 serum samples were collected, including 91 from patients with SCLC or NSCLC, 22 from patients with benign lung diseases, and 37 from healthy subjects. Enzyme-linked immunosorbent assay was used to determine serum FST levels in healthy subjects, patients with benign lung diseases and patients with lung cancers.

**Results:**

Serum FST levels in patients with LADC, SCC, LASC, LCLC, and SCLC were much higher than those in healthy subjects and in patients with lung benign disease. A ROC curve was constructed for differentiating the lung cancer from the healthy subjects and benign lung diseases. The results indicated that the area under the ROC curve (AUC) was 0.971 and 0.728 respectively. According to TNM staging, serum FST level increased significantly in patients with stage III and IV of LADC. Moreover, serum FST expression were increased in LADC patients with different TNM category. Furthermore, we found that a higher expression of serum FST was correlated with recurrence in LADC patients.

**Conclusions:**

The serum FST levels gradually increased with the rise of TNM staging and category in lung cancer patients. These data suggest that serum FST levels not only can be used in auxiliary diagnosis for lung cancer but also might be associated with the disease progression and metastasis of lung cancers.

## Background

Lung cancer is a worldwide health problem, with more than 1.8 million new cases and almost 1.6 million deaths estimated in 2012 [1, 2]. Inadequate early diagnosis is one of the major reasons for the fast-growing incidence of lung cancer in recent years, which is especially common in developing countries. Thus, exploiting new diagnostic methods is essential for extending the survival of patients with lung cancer.

Tumor biomarkers, which highly express in tumors tissues, are major indicators in auxiliary diagnosis for tumor. So far, several tumor biomarkers have been applied to the clinical diagnosis, such as AFP in hepatocellular carcinoma, CEA, and CA19-9 in colorectal carcinoma and CA125 in ovarian carcinoma [3–8]. The biomarkers closely correlating with lung cancer mainly include NSE, CEA, CA19-9, CYFRA21, SCCA and PROGRP, which the specificity and susceptibility account for 20–62% [9–11]. Since tumor biomarkers in the blood can be quickly and easily obtained in a noninvasive manner, the development of potential blood-based markers will be helpful for early diagnosis of lung cancer, monitoring of disease status, development of targeted therapies, evaluation of response to therapy and survival.

Follistatin (FST), a single chain glycoprotein, is originally isolated from the follicular fluid of ovary, which can suppress follicle-stimulating hormone (FSH) secretion from anterior pituitary cells and participate in various physiological and pathological processes [12–15]. FST widely exists in gonads and extragonadal tissues, peripheral blood and cell culture supernatant [16–19]. Serum FST levels were correlated not only with pregnancy but also with various solid tumors, including gonadal cancer, gastric cancer, hepatocellular carcinoma, basal cell carcinoma, and melanoma [20–23]. The recent studies have reported that FST was aberrantly expressed in human lung adenocarcinoma cells, suggesting that FST might be a potential biomarker for diagnosis of lung adenocarcinoma [22, 24]. However, it remains unclear whether serum FST expression is associated with lung cancer patients with different histological types, TNM staging, tumor progression, and recurrence.

In this study, we firstly investigated the association of serum FST levels with patients in two broad histological subtypes of lung cancers: small cell lung cancer (SCLC) and non-small cell lung cancer (NSCLC), and NSCLC was subdivided into lung adenocarcinoma (LADC), squamous cell carcinoma (SCC), lung adenosquamous cell carcinoma (LASC) and large cell lung cancer (LCLC). Next, we assessed FST expression in LADC according to TNM staging and category. Finally, we analyzed the relationship between serum FST expression and recurrence in LADC patients.

## Materials and methods

### Patients and healthy subjects

The subjects were chosen from both the patients with lung cancer (LC) and the patients with benign lung diseases (BLD) admitted into Tianjin Medical University Cancer Institute & Hospital between October 2014 and December 2016. All diseases were verified by pathological and cytological diagnoses. Tumor node metastasis (TNM) staging were based on the Criteria of Lung Cancer Staging from the 8th edition of the Union for International Cancer Control (UICC) and American Joint Committee on Cancer (AJCC) Cancer Staging Manual [25–27]. The healthy subjects (HSs) were selected randomly from the Physical Examination Center of Tianjin Medical University Cancer Institute & Hospital. We collected the clinical data of all the subjects, including name, age, serology, imaging (ultrasound, CT, MRI, etc.), pathology, etc. The patients with benign lung disease excluded malignant tumors, and the health subject’s imaging excluded lung disease. All subjects excluded autoimmune diseases, cardiovascular diseases, severe liver and kidney diseases, blood diseases, infectious diseases, and other malignant tumors.

### Serum sample processing

Peripheral blood was collected from each patient under an empty belly in the morning of the second day after hospitalization according to the previously described methods [21]. The serum was obtained by centrifuging at 1500*g* for 10 min at 4 °C then stored at − 80 °C 200 μL/tube separately. The control serum samples were similarly collected from the healthy subjects in the morning on the day of their routine examination.

### ELISA for serum FST

Serum FST levels in patients with LADC, SCC, LASC, LCLC and SCLC were measured by using ELISA kits (R&D Systems, Minneapolis, USA) according to the manufacturer’s instructions. Absorbance at 450 nm was measured and the serum FST levels were calculated based on the standard curve.

### Statistical methods

Data were expressed as the mean ± standard deviation and the differences among groups were compared via ANOVA. A value of *P *< 0.05 was considered statistically significant. The diagnostic performance of FST was evaluated by nonparametric receiver operating characteristic (ROC) curves, sensitivity, specificity, the area under the ROC curve (AUC), and with 95% confidence intervals (CI). The cut-off value of FST was calculated by Youden’s index, the peak point of ‘sensitivity + specificity − 1’, according to all points of a ROC curve, and served as a standard for choosing the most suitable cut-off value. Data analyses were performed by SPSS 16.0 for Windows (SPSS Inc, Chicago, IL, USA).

## Results

### Clinical characteristics of healthy subjects and patients

A total of 150 serum samples were collected, including 91 patients with lung cancer, 22 benign lung diseases patients with pulmonary tuberculosis or fibroma and 37 healthy subjects. The histological type of lung cancer was identified by a pathological expert using H&E staining. TNM staging was based on the eighth TNM staging system (8-TNM) [25–27]. Characteristics of healthy subjects, patients with benign lung diseases and patients with lung cancer were presented in Table 1. Table 2 showed the association of serum FST levels with gender and age, we found that serum FST levels had no significant correlation with gender and age. As LADC ranks first in the incidence of lung cancers [28], the category and staging of TNM in the patients with LADC were independently shown in Table 3.

**Table 1:** Characteristics of patients with lung cancer (LC), patients with benign lung diseases (BLD) and healthy subjects (HSs)

	Total (n)	Gender		Age	
		Male (n)	Female (n)	< 60 years	≥ 60 years
Healthy subjects	37	25	12	26	11
Benign lung diseases	22	10	12	18	4
Lung cancer	91	63	28	44	47
NSCLC	67	43	24	32	35
LADC	33	16	17	17	16
SCC	29	23	6	12	17
LASC	2	2	0	0	2
LCLC	3	2	1	2	1
SCLC	24	20	4	13	11
Total	150	98	52	88	62

**Table 2:** Characteristics of serum FST levels in subjects by gender and age

	Total (n)	Mean ± SD, pg/mL (male)	Mean ± SD, pg/mL (female)	Mean ± SD, pg/mL (< 60 years)	Mean ± SD, pg/mL (≥ 60 years)
Healthy subjects	37	780.89 ± 122.37	716.76 ± 85.24	746.93 ± 83.80	787.52 ± 159.68
Benign lung diseases	22	1194.48 ± 320.23	1251.53 ± 323.74	1207.00 ± 322.37	1309.28 ± 314.66
Lung cancer	91	1548.90 ± 388.54	1580.00 ± 360.68	1555.88 ± 402.03	1560.88 ± 359.06

Serum FST levels were not correlated with gender and age

**Table 3:** Category and staging of LADC according to 8-TNM

	Total (n)	Gender		Age	
		Male (n)	Female (n)	< 60 years	≥ 60 years
TNM category					
T					
T1	11	4	7	7	4
T2	17	10	7	9	8
T3	2	1	1	0	2
T4	3	1	2	1	2
N					
N0	17	10	7	7	10
N1	2	1	1	1	1
N2	12	4	8	9	3
N3	2	1	1	0	2
M					
M0	26	14	12	13	13
M1	7	2	5	4	3
TNM staging					
I	14	9	5	7	7
II	5	2	3	2	3
III	7	3	4	4	3
IV	7	2	5	4	3

### Serum levels of FST in lung cancer patients with different histological types

**Fig. 1:**
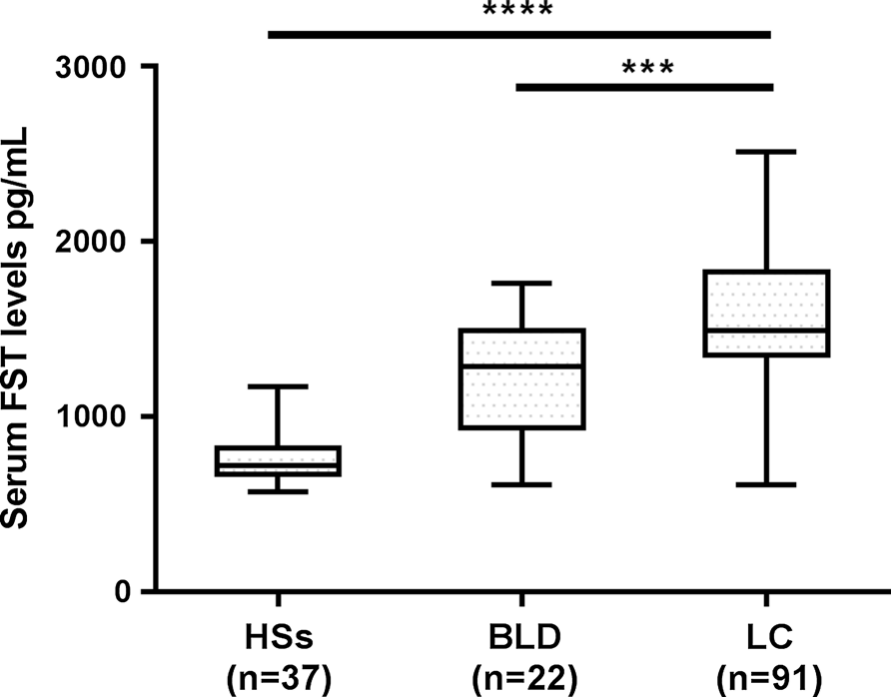
Serum FST levels in patients with lung cancer as compared with the healthy subjects and the patients with benign lung diseases. Serum FST levels (pg/mL) in the healthy subjects (HSs) (n = 37), patients with benign lung diseases (BLD) (n = 22) and patients with lung cancer (LC) (n = 91). Asterisks indicate values that are significantly different compared to that in the healthy group (***P < 0.001, ****P < 0.0001)

We firstly investigated the association between serum FST expression and the patients with lung cancer. We found that serum FST levels in patients with lung cancer were significantly higher as compared to HSs (*P *< 0.0001) and BLD (*P *< 0.001; Fig. 1).

**Fig. 2:**
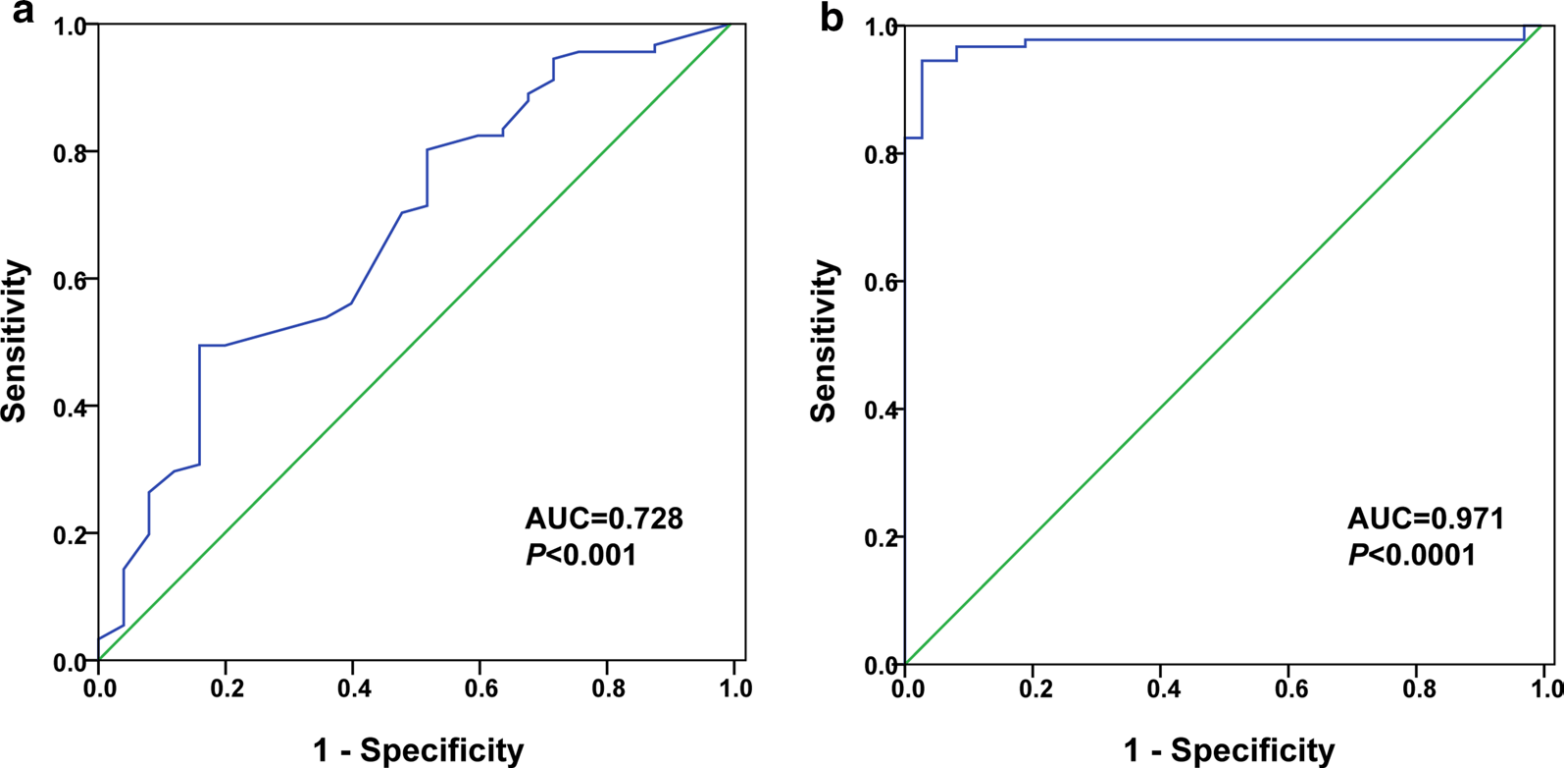
The diagnostic power of FST for lung cancer (n = 91) against the healthy subject (n = 37) and patients with benign lung disease (n = 22). **a** Power of FST in differentiating LC patients from BLD. Optimal cutoff value, where the sum of sensitivity and specificity was maximum, were 1509.55 pg/mL. **b** Power of FST in differentiating LC patients from HSs. The optimal cutoff values were 970.74 pg/mL. ROC, receiver operating characteristic; AUC, area under the curve

ROC curves and the area under the curve (AUC) were used to assess the performance of the serum FST level as a biomarker for lung cancer diagnosis. The results showed that the AUC was 0.728 (95% confidence interval 0.636–0.807; *P *< 0.001, to see Fig. 2) in differentiating LC patients from BLD, with the optimal cut-off value of 1509.55 pg/mL. The AUC was 0.971 (95% confidence interval 0.926–0.993; *P *< 0.0001, to see Fig. 2) in differentiating LC patients from HSs, with the optimal cut-off value of 970.74 pg/mL.

We next calculated the sensitivity and specificity of serum FST level in patients with different histological types of lung cancer using 1509.55 pg/mL and 970.74 pg/mL as the cut-off value respectively. We found that there were significant differences in patients with LC patients as compared to HSs and BLD. In contrast, no significant differences were found among different histological types of lung cancer (Table 4).

**Table 4:** Serum FST levels in healthy subjects and patients with benign diseases or lung cancer

Subjects	n	FST mean ± SD (range), pg/mL	LC patients vs BLD patients		BLD/LC patients vs HSs	
			Sensitivity	Specificity	Sensitivity	Specificity
Healthy subjects	37	760.09 ± 115.65 (570.88–1170.24)	–	–	–	–
Benign lung diseases	22	1225.60 ± 323.40 (608.36–1763.76)^#^	–	–	68.18%	97.30%
Lung cancer	91	1558.47 ± 380.45 (608.36–2513.85)^#^**	45.45%	90.91%	94.51%	97.30%
LADC	33	1526.71 ± 305.68 (877.29–2241.09)^#^**	45.45%	90.91%	96.97%	97.30%
SCC	29	1569.30 ± 411.57 (608.36–2377.47)^#^**	44.83%	90.91%	93.10%	97.30%
LASC	2	1559.17 ± 136.38 (1422.81–1695.57)	50%	90.91%	100.00%	97.30%
LCLC	3	1877.41 ± 362.26 (1422.81–2309.28)	66.67%	90.91%	100.00%	97.30%
SCLC	24	1549.11 ± 428.23 (608.36–2513.85)^#^**	58.33%	90.91%	91.67%	97.30%

^#^*P *< 0.01 compared with healthy group; * *P *< 0.05, ** *P *< 0.01, compared with benign disease group

Taken together, these results suggested that expression of serum FST seemed likely to have a potential diagnostic value in patients with lung cancer.

### Serum levels of FST in patients with LADC according to TNM staging

Since LADC is the most common histological type of lung cancer with a classical TNM staging [28], we further evaluated serum FST expression in patients with LADC according to the TNM staging. As shown in Table 5, serum FST levels significantly increased in all stage LADC, especially in stage III and IV patients as compared with HSs, BLD and the patients with I–II stage.

**Table 5:** Serum FST levels in patients with LADC according to TNM staging

	n	Mean ± SD, pg/mL	Minimum–maximum, pg/mL
Healthy subjects	37	760.09 ± 115.65	570.88–1170.24
Benign diseases	22	1225.60 ± 323.40^#^	608.36–1763.76
LADC	33		
I	14	1404.69 ± 245.02^#^	877.29–1900.14
II	5	1409.18 ± 199.51^#^	1150.05–1763.76
III	7	1578.68 ± 276.90^#^*	1150.05–2104.71
IV	7	1802.73 ± 311.12^#^**^S^	1150.05–2241.09

^#^*P *< 0.01 compared with the healthy group; * *P *< 0.05, ** *P *< 0.01, compared with benign disease group; ^S^*P *< 0.05, compared with I and II stage group

### Serum levels of FST in patients with LADC according to T category

Serum FST levels were evaluated in patients with LADC according to T category in 8-TNM staging system. The results showed that serum FST levels were significantly increased in patients with T1 and T2 subgroups of LADC (T3 and T4 subgroups had not carried out the statistical analysis because of only 2–3 samples), compared with those in the healthy subject group and lung benign disease group, but there were no significant differences among T categories (Table 6).

**Table 6:** Serum FST levels in patients with LADC according to T category

	n	Mean ± SD, pg/mL	Minimum–maximum, pg/mL
Healthy subjects	37	760.09 ± 115.65	570.88–1170.24
Benign diseases	22	1225.60 ± 323.40^#^	608.36–1763.76
LADC	33		
T1	11	1492.72 ± 378.65^#^*	877.29–2241.09
T2	17	1474.96 ± 237.98^#^*	1150.05–1900.14
T3	2	1797.86 ± 170.47	1627.38–1968.33
T4	3	1763.76 ± 192.87	1491.00–1900.14

^#^*P *< 0.01 compared with the healthy group; * *P *< 0.05, ** *P *< 0.01, compared with benign disease group

### Serum levels of FST in patients with LADC according to N category

Similarly, serum FST levels were examined in patients with LADC classified as N category in 8-TNM staging system. Serum FST expression was increased in patients with different N category of LADC, and especially, significantly higher in patients with N0 and N2 category (N1 and N3 subgroups were not analyzed because of the above reasons), compared with those in healthy subject group, lung benign disease group (Table 7).

**Table 7:** Serum FST levels in patients with LADC according to N category

	n	Mean ± SD, pg/mL	Minimum–maximum, pg/mL
Healthy subjects	37	760.09 ± 115.65	570.88–1170.24
Benign diseases	22	1225.60 ± 323.40^#^	608.36–1763.76
LADC	33		
N0	17	1375.80 ± 214.15^#^	877.29–1763.76
N1	2	1525.10 ± 102.28	1422.81–1627.38
N2	12	1672.84 ± 329.78^#^**^S^	1150.05–2241.09
N3	2	1934.24 ± 34.09	1900.14–1968.33

^#^*P *< 0.01 compared with the healthy group; * *P *< 0.05, ** *P *< 0.01, compared with benign disease group

^S^*P *< 0.05 compared with group N0

### Serum levels of FST in patients with LADC according to M category

Furthermore, we assessed serum FST levels in patients with LADC classified as M category in 8-TNM staging system. A higher level of serum FST expression was found in patients with M0 and M1 category of LADC, and especially, significantly increase in patients with the M1 category, compared with those in the healthy subject group, lung benign disease group and patients with M0 group of LADC (Table 8).

**Table 8:** Serum FST levels in patients with LADC according to M category

	n	Mean ± SD, pg/mL	Minimum–maximum, pg/mL
Healthy subjects	37	760.09 ± 115.65	570.88–1170.24
Benign diseases	22	1225.60 ± 323.40^#^	608.36–1763.76
LADC	33		
M0	26	1452.39 ± 257.88^#^*	877.29–2104.71
M1	7	1802.73 ± 311.12^#^**^S^	1150.05–2241.09

^#^*P *< 0.01 compared with the healthy group; * *P *< 0.05, ** *P *< 0.01, compared with benign disease group; ^S^ *P *< 0.05, compared with the M0 group

### Serum FST levels in patients with recurrent lung cancer

Finally, serum FST levels were evaluated in patients with recurrent lung cancer. The result showed that in the diagnosed patients with recurrent, serum FST levels are much higher than those in the healthy subject group (Table 9).

**Table 9:** Serum FST levels in patients with recurrent lung cancer

	N	Mean ± SD, pg/mL	Minimum–maximum, pg/mL
Healthy subjects	37	760.09 ± 115.65	570.88–1170.24
Recurrent lung cancer group	28	1209.16 ± 312.20^#^	601.36–2613.85

^#^*P *< 0.01 compared with the healthy group

## Discussion

Lung cancer is the most frequent cancer diagnosed and the leading cause of mortality in the world. Reductions in lung cancer mortality can be attained through treatment, especially if the disease is diagnosed at a stage where curative therapy is possible. Thus, it is urgent for finding new molecular biomarkers for early diagnosis of lung cancer, monitoring of disease status, development of targeted therapies, evaluation of response to therapy and survival [3].

FST is a monomeric, cysteine-rich polypeptide which suppresses pituitary FSH release in a similar manner to inhibin [13, 29]. Subsequent discovers indicate that this molecule can also play a variety of roles in several reproductive and nonreproductive systems as potent tissue regulators in the gonad, pituitary gland, pregnancy membranes, vasculature, and liver [30]. Recent studies suggest that FST, as a stress responsive protein, plays a protective role under a variety of stresses [31]. In addition, inactivation of hepatic FST may contribute to improve glucose tolerance and alleviate hyperglycemia [32, 33].

Accumulating evidence indicates that FST has been implicated in the development and progression of solid tumours [23]. Overexpression of FST was found in several human tumors, including gastric cancer [34], ovarian cancer [35], prostate cancer [36], basal cell carcinoma [37] and hepatocellular carcinoma [38]. Several recent studies revealed the closed relationship between FST and breast cancer, one of these studies by Zabkiewicz et al. showed that FST overexpression appears to promote breast cancer in vitro proliferation and reduce invasiveness [39–41]. Furthermore, FST plays also a role in angiogenesis and metastasis of solid tumours. The effect of FST on tumour angiogenesis seems to be complex, some observations in both lung- and liver-derived tumours are strongly suggestive of FST inhibiting tumour angiogenesis [42], but other evidence shows FST may have a promotory effect on tumour angiogenesis [43, 44]. Additionally, some studies support the role of FST in controlling tumor metastasis [40, 42, 45, 46]. Very recently, Seachrist et al. [47] found FST is a metastasis suppressor in a mouse model of HER2-positive breast cancer.

FST has shown strong promise as a diagnostic or prognostic marker for solid tumours. Some studies reported that serum FST levels were significantly increased in patients with ovarian cancer [21], hepatocellular cancer [48], and breast cancer [39, 49]. In the previous study, we have reported serum FST overexpression in lung adenocarcinoma [22]. However, the prognostic value of FST in the serum of lung patients with different types, TNM staging, and recurrent lung cancer remains poorly investigated.

In this study, we firstly examined serum FST levels in lung cancer patients with different histological types. We found that serum FST levels in patient group with lung cancer were significantly higher than those in the healthy control group and lung benign disease group. ROC analysis revealed that when compared LC with HSs and BLD, the serum FST levels provided a diagnosis efficacy with AUC of 0.971 and 0.728 respectively, indicating that serum FST seemed likely to have a potential role in lung cancer diagnosis. Since LADC ranks first in the incidence of lung cancers with all histological types and the studies on individualized LADC treatments are gradually intensified, we further observed the correlation of serum FST levels with LADC according to the TNM staging. Our data showed that serum FST levels had a much higher expression in patients with stage III and IV LADC. Simultaneously, our results also showed that serum FST levels increased significantly in patients with different T category of LADC, but there was no significant difference among the T categories. Moreover, serum FST levels were also elevated in patients with LADC according to N and M categories. Notably, we found that serum FST was elevated in the diagnosed patients with recurrent as compared to the healthy subjects. Taken together, the above results indicated that serum FST levels modestly reflected the disease progression and metastasis of lung cancers.

To date, the potential mechanism for an increase of serum FST levels in human carcinogenesis is not clear, a possible mechanism of FST overexpression may represent a unique strategy of tumors to overcome the inhibitory action of activin by decreasing its local bioavailability [50].

Although our data has shown a close relationship between FST expression and lung cancer with different histologic types, TNM staging and disease recurrence after surgery, thus suggesting the potential of FST as a biomarker for lung cancer diagnosis, we are aware that the sample size in this cohort is rather small, which limits the power of multivariate analyses, therefore, further validation by larger scale prospective trials is needed. Another limitation of our study is the use of one testing methodology, i.e., serum FST levels measurement by ELISA, it needs to be further corroborated by optimal tissue-based analysis of FST expression in lung cancer tissues. Furthermore, although rigorous screening in this experiment has been performed, future study needs to take into consideration of the possibility that the patients were previously treated, because local inflammatory response to therapy could also contribute to an increases of serum FST levels.

## Conclusions

In summary, a significant increase of serum FST levels in the patients with lung cancer and those with recurrent lung cancer is closely related to the clinical staging of tumors. Therefore, determination of serum FST levels not only can be used in the auxiliary clinical diagnosis of lung cancer but also might be associated with tumor progression and metastasis.

## Abbreviations

FST: follistatin
FSH: follicle-stimulating hormone
AFP: alpha-fetoprotein
CEA: carcinoembryonic antigen
CA19-9: cancer antigen 19-9
CA125: cancer antigen 125
NSE: neuron-specific enolase
CYFRA21: cytokeratin fragment 21
TNM: tumor node metastasis
LC: lung cancer
BLD: benign lung diseases
HSs: healthy subjects
SCLC: small cell lung cancer
NSCLC: non-small cell lung cancer
LADC: lung adenocarcinoma
SCC: squamous cell carcinoma
LASC: lung adenosquamous cell carcinoma
LCLC: large cell lung cancer
ROC: receiver operating characteristic
AUC: area under the curve
CI: 95% confidence intervals
ELISA: enzyme-linked immunosorbent assay
CT: computed tomography
8-TNM: eighth TNM staging system
UICC: International Cancer Control
AJCC: American Joint Committee on Cancer
